# Changes in real-life practice for hepatocellular carcinoma patients in the Republic of Korea over a 12-year period: A nationwide random sample study

**DOI:** 10.1371/journal.pone.0223678

**Published:** 2019-10-17

**Authors:** Beom Kyung Kim, Do Young Kim, Kwang-Hyub Han, Jinsil Seong

**Affiliations:** 1 Division of Gastroenterology, Department of Internal Medicine, Yonsei University College of Medicine, Seoul, Republic of Korea; 2 Yonsei Liver Center, Severance Hospital, Seoul, Republic of Korea; 3 Department of Radiation Oncology, Yonsei University College of Medicine, Seoul, Republic of Korea; Seoul National University College of Pharmacy, REPUBLIC OF KOREA

## Abstract

**Backgrounds & aims:**

Comprehensive analyses through nationwide hepatocellular carcinoma (HCC) registries are important to understand health care issues. We assessed changes in real-life practice for HCC over a long time period.

**Methods:**

The Korean Liver Cancer Association and the Korean Central Cancer Registry jointly established the nationwide cohorts of newly diagnosed HCC patients between 2003 and 2005 and between 2008 and 2014. According to sorafenib reimbursement in the Republic of Korea (January 2011), patients were divided into early (E-Cohort: 2003~2010) and late (L-Cohort: 2011~2014) cohorts.

**Results:**

L-Cohort (n = 4776) comprised patients with older age (60.8 vs. 58.3 years), higher proportions of patients with well-preserved liver function (75.6% vs. 68.2%) and non-viral etiologies (28.6% vs. 19.4%), and lower proportion of patients with Barcelona Clinic Liver Cancer [BCLC] 0~A stage (46.2% vs. 53.9%) than E-Cohort (n = 8203) (all p<0.05). Proportions of patients undergoing curative treatments were higher in L-Cohort than in E-Cohort (55.0% vs. 35.1%, 23.2 vs. 11.3%, and 17.3% vs. 9.6% in BCLC 0A, B, and C stages, respectively; all p<0.05). Accordingly, compared with that in E-Cohort, overall survival in L-Cohort significantly improved in patients with BCLC 0~A, B, and C stages (all p<0.05). As first-line treatment, 62.4% underwent locoregional treatments (LRTs), whereas only 9.7% received sorafenib, among BCLC stage C patients in L-Cohort.

**Conclusions:**

For the past 12 years, curative treatments became more widely available to BCLC 0~A, B, and C stage patients, generally improving prognosis. Despite sorafenib reimbursement, LRTs remain the mainstay of first-line treatment for BCLC C stage patients.

## Introduction

Hepatocellular carcinoma (HCC) is the sixth commonest cancer worldwide [1]. Global variation in HCC incidences largely depends on the underlying risk factors dominant in each geographic area, including chronic hepatitis B virus (HBV) or hepatitis C virus (HCV) infection, alcohol intake, aflatoxin exposure, and non-alcoholic steatohepatitis [2,3]. In East Asia and Africa, the largest proportion of HCC occurs because of chronic HBV infection, whereas in Western countries, chronic HCV infection appears to be the major risk factor. In the Republic of Korea, chronic HBV infection is the predominant etiology of HCC, although its overall prevalence has declined gradually, primarily owing to a national vaccination program implemented in 1995 [4]. However, according to the 2005 Korean National Health and Nutrition Examination Survey, the rate of positive serum hepatitis B surface antigen among people aged ≥10 years was still as high as 4.8% for males and 3.0% for females [5]. Furthermore, although the overall liver-related mortality has markedly improved over several decades owing to substantial advances in antiviral treatments, the number of patients at risk of developing HCC has paradoxically increased, thereby increasing the medical and socioeconomic burden of HCC [6]. The age-standardized incidence rate of HCC in the Republic of Korea was 19.9/100,000 in 2014, making it the seventh commonest cancer [7].

Despite this increase in incidence, considerable positive changes have been made in medical and socioeconomic fields in terms of treatment modalities and prognosis among Korean HCC patients. In particular, sorafenib was approved in 2008 for advanced stage HCC patients and has been reimbursed in clinical practice since 2011. Sorafenib is the first molecular targeted agent to block the Raf/MEK/ERK pathway by inhibiting Raf serine/threonine kinase and the upstream receptor tyrosine kinases that promote angiogenesis, such as vascular endothelial growth factor receptor (VEGFR)-2, VEGFR-3, platelet-derived growth factor receptor β, and kit [8]. Thereafter, various kinds of efforts to improve prognosis have been performed [9–12]. Furthermore, vigorous therapeutic approaches and improved liver function through the development of antivirals or general supportive care could positively affect the overall prognosis [13,14].

To date, most clinical studies of HCC in the Republic of Korea have been conducted using cohorts from at most a few hospitals, which were inevitably subject to selection bias. To collect unbiased data on clinical characteristics, treatment patterns, and survival of HCC patients in the Republic of Korea, the Korean Liver Cancer Association (KLCA) established a nationwide HCC registry alongside the statutory Korean Central Cancer Registry (KCCR).

Based on this nationwide registry data, we aimed to comprehensively analyze changes in clinical characteristics, treatment patterns, and survival among Korean HCC patients across a long time period.

## Materials and methods

### Patients

In total, 15,098 patients were randomly selected from newly registered HCC cases in KCCR between 2003 and 2005 and between 2008 and 2014. In the Republic of Korea, patients diagnosed with cancer receive additional economic assistance through a medical reimbursement policy when they are registered at KCCR; hence, almost all patients with incident cancers are enrolled in the registry. HCC was diagnosed based on histological or radiological evaluation with reference to the American Association for the Study of Liver Disease, European Association for the Study of the Liver, or KLCA guidelines [15–18]. Patients were divided into early (the E-Cohort: between 2003 and 2010) and late (the L-Cohort: since 2011) cohorts according to the time of sorafenib reimbursement in the Republic of Korea (January 2011).

Data regarding clinical characteristics such as age; gender; date of diagnosis; etiology; Child-Pugh class; serum laboratory tests, including albumin, total bilirubin, prothrombin time, creatinine, and sodium; tumor characteristics such as maximum tumor size, tumor number, presence of portal vein invasion, regional lymph node involvement, and extrahepatic spread; and first-line treatment modality were collected. Tumor stage was categorized using the Barcelona Clinic Liver Cancer (BCLC) staging system [19]. Treatment modalities were classified into curative treatments (liver transplantation, resection, and local ablation) and palliative treatments (trans-hepatic arterial therapy, external beam radiotherapy, systemic chemotherapy, and others). Patients with any missing data about age, gender, Child-Pugh class, BCLC stages, and follow-up were excluded.

The study protocol was consistent with the ethical guidelines of the 1975 Declaration of Helsinki and was approved by the Institutional Review Board (IRB). Primarily owing to a retrospective nature of the study, the IRB waived the requirement for informed consent. Furthermore, all data were fully anonymized before authors accessed them.

### Statistical analysis

Variables are expressed as the mean ± standard deviation, median (interquartile range), or number (%), as appropriate. Differences among continuous and categorical variables were assessed for statistical significance using the Student’s *t-*test (or Mann-Whitney test, if appropriate) and chi-squared test (or Fisher’s exact test, if appropriate). Overall survival (OS) was defined as the time interval from the date of diagnosis to the date of death or last follow-up and was calculated using the Kaplan-Meier analysis with a comparison by log-rank test.

If necessary, multivariate analysis using the Cox-proportional hazard model was used to identify independent prognostic factors and calculate adjusted hazard ratios (HRs). Furthermore, if appropriate, to reduce the effect of selection bias and potential confounders between groups, differences in baseline characteristics were adjusted through inverse probability treatment weighting (IPTW) analysis using propensity scores calculated through logistic regression analysis.

Statistical analysis was performed using IBM^®^ SPSS^®^ Statistics Version 23.0 (IBM Corporation, Armonk, NY, US), R (V.3.4.4, http://cran.r-project.org/), and SAS (version 9.4, SAS Inc., Cary, NC, USA). Two-sided p-values <0.05 were considered to indicate statistical significance.

## Results

### Patient characteristics

In total, 12,979 HCC patients were included. The baseline clinical characteristics of the two groups are presented in Table 1. The L-Cohort (n = 4776) comprised older patients (60.8 years vs. 58.3 years) and had higher proportions of patients with well-preserved liver function (Child-Pugh class A; 75.6% vs. 68.2%) and non-viral etiologies (28.6% vs. 19.4%) and a lower proportion of patients with early stage HCC (46.2% vs. 53.9%) with statistical significances (all p<0.001), compared to the E-Cohort (n = 8203).

**Table 1:** Baseline clinical characteristics between the E-Cohort and the L-Cohort.

	E-Cohort (n = 8203)	L-Cohort (n = 4776)	p-value
**Age, years**	58.3 ± 10.9	60.8 ± 11.3	<0.001
**Male gender**	79.3%	79.9%	0.414
**BCLC stages**			<0.001^a^
0	652 (7.9%)	294 (6.2%)	
A	3772 (46.0%)	1912 (40.0%)	
B	875 (10.7%)	604 (12.6%)	
C	2287 (27.8%)	1663 (34.8%)	
D	621 (7.6%)	303 (6.4%)	
**Etiology**			<0.001^b^
HBV	5013 (61.1%)	2804 (58.7%)	
HCV	858 (10.5%)	549 (11.5%)	
HBV+HCV	95 (1.2%)	58 (1.2%)	
Alcohol	819 (10.0%)	630 (13.2%)	
Others	770 (9.4%)	735 (15.4%)	
Unknown	648 (7.8%)	0 (0.0%)	
**Child Pugh class**			<0.001^c^
A	5594 (68.2%)	3610 (75.6%)	
B	2037 (24.8%)	937(19.6%)	
C	572 (7.0%)	229 (4.8%)	

Values were expressed as mean ± standard deviation or no.(%).

^a^BCLC stages 0 and A vs. B, C, and D

^b^Non-viral etiologies vs. the remainder

^c^Child Pugh class A vs. B and C

**Abbreviations**: BCLC, Barcelona clinic liver cancer; HBV, hepatitis B virus; HCV, hepatitis C virus

### First-line treatment modalities

The applied first-line treatment modalities in the E-Cohort and L-Cohort are described in Table 2. Overall, the L-Cohort had a significantly higher proportion of patients receiving curative modalities as first-line treatment (35.2% vs. 23.6%) than the E-Cohort. Among patients with BCLC stages 0~A, B, and C, the proportions of patients undergoing curative treatments were higher in the L-Cohort than in the E-Cohort (55.0% vs. 35.1%, 23.2% vs. 11.3%, and 17.3% vs. 9.6%, respectively; all p < 0.001). The detailed first-line treatment modalities according to BCLC stage in the E-Cohort and L-Cohort are depicted in S1 Fig and S1 Table.

**Table 2:** The first-line treatment modalities between the E-Cohort and the L-Cohort.

	E-Cohort (n = 8203)	L-Cohort (n = 4776)	p-value
**Treatment modality**			<0.001
Resection	1098 (13.4%)	1049 (22.0%)	
Liver transplant	62 (0.8%)	60 (1.3%)	
Local ablation	774 (9.4%)	570 (11.9%)	
Trans-hepatic arterial therapy	4175 (50.9%)	1982 (41.5%)	
Systemic chemotherapy	128 (1.6%)	257 (5.4%)	
External beam radiotherapy	88 (1.1%)	72 (1.5%)	
Best supportive care	628 (7.6%)	674 (14.1%)	
Unknown	1520(15.2%)	112 (2.3%)	

Values were expressed as mean ± standard deviation or no.(%).

**Abbreviations**: BCLC, Barcelona clinic liver cancer; HBV, hepatitis B virus; HCV, hepatitis C virus

The adherence rates to BCLC treatment guidelines for each BCLC stage are summarized in Table 3. Adherence in the BCLC stages 0~A was higher in the L-Cohort than in the E-Cohort (55.0% vs. 35.1%; p < 0.001), whereas the proportion of those treated with trans-hepatic arterial therapy significantly decreased, from 53.1% to 39.1% (p < 0.001). In patients with BCLC stage B HCC, the proportion of patients undergoing trans-hepatic arterial therapy was lower in the L-Cohort than in the E-Cohort (58.8% vs. 65.5%; p = 0.009). Because sorafenib started to be reimbursed in January 2011 in the Republic of Korea, the L-Cohort had a significantly higher proportion of patients treated with sorafenib. However, despite the sorafenib reimbursement policy, only a small proportion (9.7%) of patients with advanced stage HCC underwent sorafenib treatment as first-line therapy. Among patients with BCLC C stage in the L-Cohort, 62.4% (n = 1037) received locoregional treatments (LRTs) as first-line therapy (resection, n = 208; liver transplant, n = 12; local ablation, n = 67; trans-hepatic arterial therapy, n = 692; external beam radiotherapy, n = 58).

**Table 3:** Adherence rates of BCLC treatment guideline in each BCLC stage in the E-Cohort and the L-Cohort.

BCLC stage	E-Cohort (n = 8203)	L-Cohort (n = 4776)	
**0~A**	1552 (35.1%)	1213 (55.0%)	<0.001
**B**	573 (65.5%)	355 (58.8%)	0.009
**C**	30 (1.3%)	162 (9.7%)	<0.001
**D**	133 (21.4%)	145 (47.5%)	<0.001

**Abbreviations:** BCLC, Barcelona clinic liver cancer

### Survival outcomes

**Fig 1:**
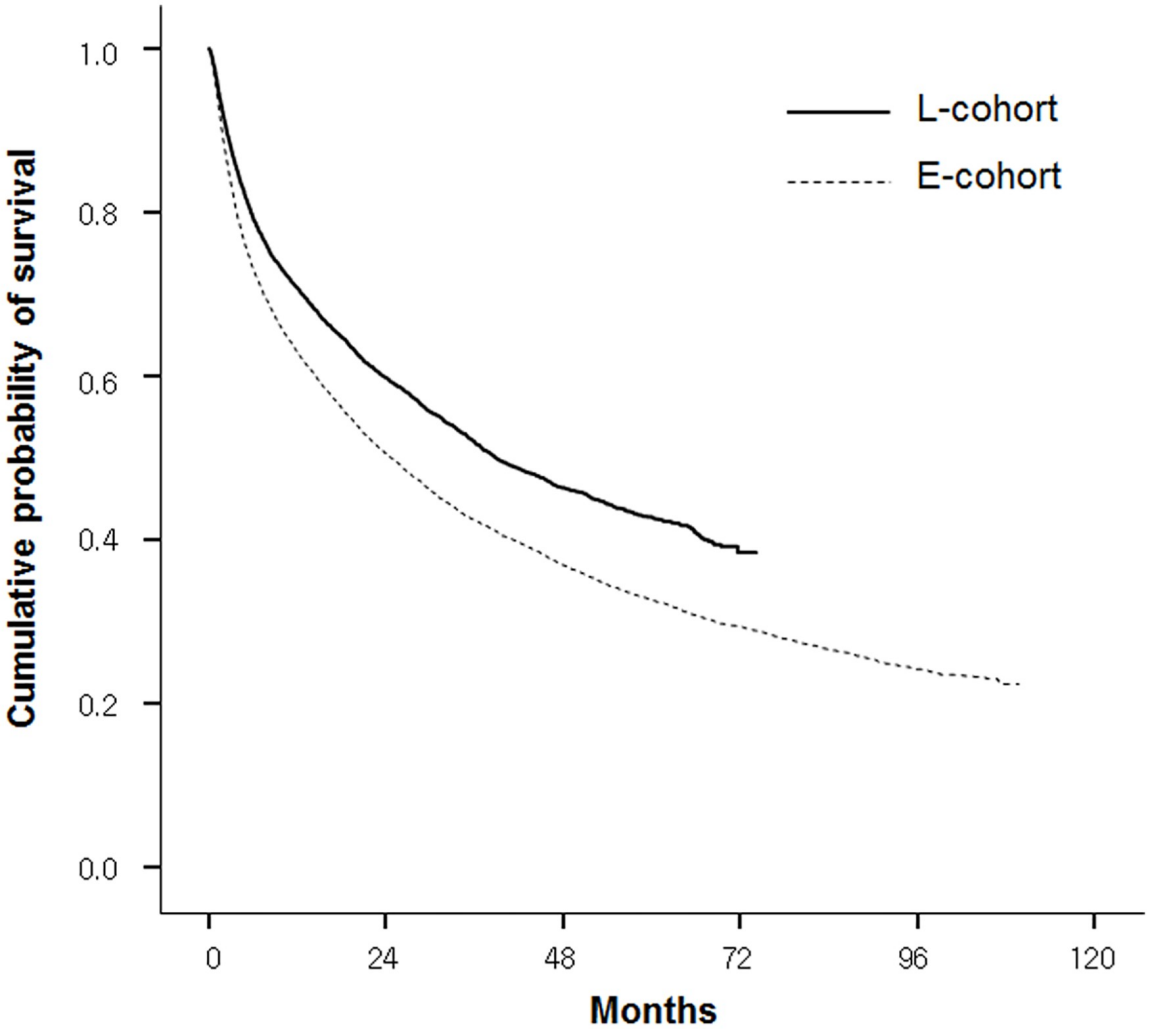
Kaplan-Meier analysis of overall survival between the E-Cohort and L-Cohort.

The median OS and the 5-year survival rate were significantly higher in the L-Cohort (38.9 months, 95% confidence interval [CI] 35.8–41.9 and 42.7%, respectively) than in the E-Cohort (24.8 months, 95% CI 23.5–26.2 and 32.8%, respectively) (p < 0.001) (Fig 1). The crude mortality rates within 1 year after diagnosis were 37.4% in the E-cohort and 29.4% in the L-cohort.

**Fig 2:**
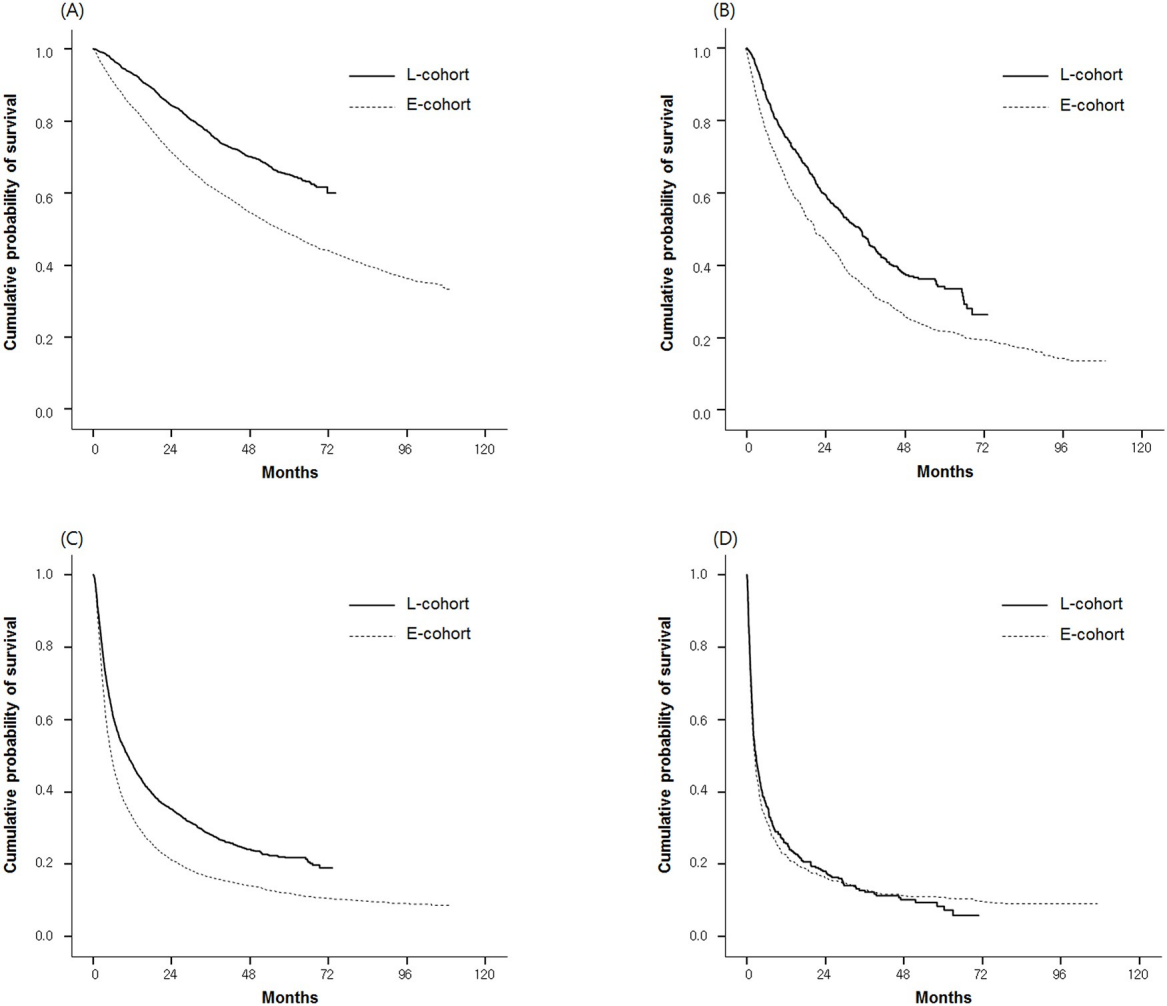
Kaplan-Meier analysis of overall survival between the E-Cohort and L-Cohort when stratified by BCLC stage 0~A (A), B (B), C (C), and D (D).

When stratified by BCLC stage, the median OS in the L-cohort was significantly higher than that in the E-Cohort for BCLC stage 0~A patients (not reached vs. 57.3 months [95% CI 30.4–54.1]; p < 0.001), stage B (34.3 [95% CI 30.3–38.4] vs. 20.9 [95% CI 18.3–23.6] months; p < 0.001), and stage C (10.5 [95% CI 9.2–11.9] vs. 5.5 [95% CI 5.1–5.9] months; p < 0.001) (Fig 2A, 2B and 2C). However, in BCLC stage D patients, the median OS did not improve in the L-Cohort compared with that in the E-Cohort (2.8 [95% CI 2.0–3.6] vs. 2.3 [95% CI 1.9–2.6] months; p = 0.495) (Fig 2D). Independent predictors affecting the OS were also assessed (S2 Table).

**Fig 3:**
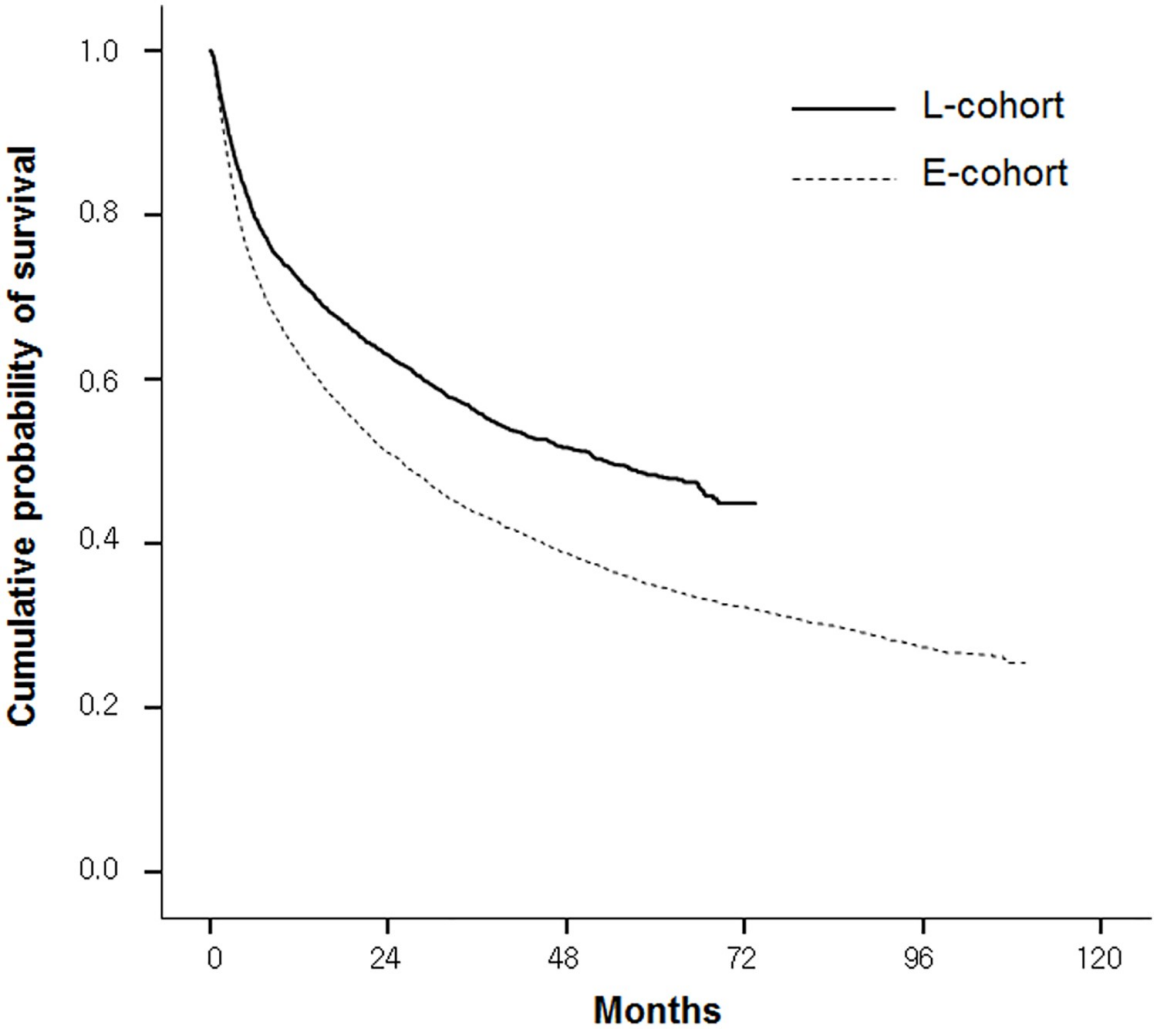
Kaplan-Meier analysis of overall survival between the E-Cohort and L-Cohort among patients with HBV-related HCC.

Furthermore, because nucleos(t)ide analogs for chronic HBV infection have been eligible for life-long reimbursement since 2010, we analyzed their effect on survival outcomes. Among patients with HBV-related HCC, the L-Cohort (n = 2862) had a significantly longer median OS than the E-Cohort (n = 5108) (53.4 months [95% CI 47.5–59.3] vs. 25.7 months [95% CI 23.8–27.6]; p < 0.001), with an adjusted HR of 0.752 (95% CI 0.704–0.804; p < 0.001) (Fig 3).

### Subgroup analysis for comparison of OS according to first-line treatment modality

Except for BCLC stage 0~A HCC patients, other treatment modalities beyond the recommended practice guidelines have previously been applied widely. Hence, we conducted subgroup analyses for comparison of OS according to first-line treatment modality.

Trans-hepatic arterial therapy is the recommend treatment modality for BCLC stage B patients. The OS of patients undergoing trans-hepatic arterial therapy (27.9 months [95% CI 25.5–30.4]) was significantly shorter than that of those undergoing other LRTs, including curative treatments (80.1 months [95% CI 60.1–100.3], p < 0.001), with an adjusted HR of 0.512 (95% CI 0.396–0.662, p < 0.001) (S2 Fig).

Furthermore, because LRTs remained the mainstay of first-line treatment for BCLC stage C patients, despite the sorafenib reimbursement policy, we compared OS according to treatment modalities (sorafenib vs. LRTs) in this patient subgroup. Since 2008, the year of sorafenib approval for HCC in the Republic of Korea, a total of 1326 patients had BCLC stage C disease with Child-Pugh class A liver function. The patients treated with LRTs (n = 1219) had better OS than those treated with sorafenib (n = 107) (23.3 months [95% CI 20.5–26.2] vs. 4.8 months [95% CI 3.5–6.1]; p < 0.001), with an adjusted HR of 0.769 (95% CI 0.611–0.969; p = 0.026) (S3 Fig). Subsequent IPTW analysis confirmed these results (20.1 months [95% CI 17.8–23.6] vs. 6.0 months [95% CI 4.2–8.7]; p < 0.001), with an HR of 0.370 (95% CI 0.315–0.433, p < 0.001).

## Discussion

To our knowledge, this is the first study to assess clinical characteristics, treatments, and survival in terms of chronological changes after reimbursement of sorafenib in the Republic of Korea using the Korean nationwide HCC registry. Our results provide insight into several important health care issues in HCC patients not only in the Republic of Korea but also in other countries with similar situations. Furthermore, from this study, the recent epidemiological data might be achieved in detail; for instance, among HBV-related HCC cases (except HBV/HCV co-infection) diagnosed since 2011, the proportion of very-early, early, and advanced HCC cases were 6.6%, 41.0%, and 52.4%, respectively.

For the past 12 years, we noticed major changes in the demographic characteristics of Korean HCC patients. First, the proportion of HBV-related HCC cases in the overall HCC population significantly decreased during this period. This is most likely because nucleos(t)ide analogs for chronic HBV infection have been eligible for life-long reimbursement since 2010 in the Republic of Korea [4,20–22]. Furthermore, because long-term nucleos(t)ide analogs can prevent the deterioration of liver function in patients with chronic HBV infection, the L-Cohort had a higher proportion of patients with well-preserved liver function than the E-Cohort (78.5% vs. 69.2%, p < 0.05). This could have contributed to better OS among patients with HBV-related HCC in the L-Cohort than in the E-Cohort. During the same period, the proportion of HCC cases with etiologies other than chronic viral hepatitis and alcohol (e.g. non-alcoholic fatty liver disease) increased from 9.4 to 15.4% in the overall HCC population, which is closely associated with a gradual increase in the prevalence of metabolic diseases [23]. The prevalence of diabetes in the Republic of Korea increased from 8.6% to 11.0% from 2001 to 2013, and the prevalence of adult obesity, the most important predisposing factor of diabetes, also increased from 29.2% to 31.8% in that time period [24]. Unlike HBV-related HCC, the proportion of HCV-related HCC remained stable during our study period. As the interferon-free regimens based on potent direct-acting antivirals became popular for patients with chronic HCV infection in 2015, the epidemiological changes in the near future should be carefully assessed. Another major epidemiological issue shown in the present study was that the early detection rate of HCC did not improve, despite the ongoing National Cancer Screening Program. Approximately half of the HCC patients were diagnosed at intermediate to advanced stages. There are several possible explanations. First, the proportion of patients with HCC by non-viral etiologies significantly increased, who were much less likely to undergo periodic HCC surveillance. Thus, their HCC stages tended to belong to intermediate to advanced stage rather than early stage. Furthermore, the low screening rate (<50% in 2015) for so called high-risk population might contribute to this phenomenon. Therefore, considering the proven benefit of HCC screening [25], more vigorous efforts need to be urgently made toward improving the implementation of HCC screening in clinical practice in the Republic of Korea [26].

In terms of first-line treatment modalities, curative treatments have become more widely available for patients with BCLC stages 0~A to stage C over the past 12 years in the Republic of Korea, generally leading to better prognosis. Within the same BCLC stage, patients who receive therapy with curative intent had better survival than those who receive palliative therapy [27]. Nevertheless, trans-hepatic arterial therapy was performed as first-line therapy for up to 40% of early stage HCC patients in the L-Cohort. This phenomenon was most likely because of the belief that trans-hepatic arterial therapy, provided that compact lipiodol deposition was achieved, might be an effective alternative for BCLC stage 0~A HCC patients when local ablation is not technically feasible [28,29].

Because the majority of HCC patients have a long-standing chronic liver disease, treatment needs to be highly individualized, given the challenges posed by the underlying liver dysfunction and the technical issues during the therapeutic process, as well as the tumor burden. Accordingly, as in other solid malignancies, a multidisciplinary approach is preferable to provide the optimized therapy for each patient, and some positive results had been reported in terms of treatment compliance and survival outcomes [30–32]. Such a practice trend might in part contribute to the increase in the proportion of BCLC stage B and C patients undergoing curative treatments over the past 12 years. We also conducted subgroup analyses to compare OS according to the first-line treatment modality. We found LRTs, including curative treatments, improved prognosis over trans-hepatic arterial therapy for BCLC stage B HCC patients, and LRTs improved survival over sorafenib for BCLC stage C HCC patients. Because we completely acknowledge the potential selection bias when clinicians decided the treatment modality, subsequent adjusted analyses using multivariate Cox regression and IPTW analysis were performed, which confirmed the reproducibility of these results. These findings are in agreement with those of Sangiovanni et al. [31], who also reported consistent trends toward a better prognosis in BCLC stage B and C HCC patients treated with so-called “upward treatment migration.”

This study had some limitations. First, approximately 14% of the overall cohort was unavailable owing to missing data of critical parameters. Nevertheless, because it is a nationwide, random cohort study with a long-term follow-up, which has a minimal selection bias, we believe it provides a comprehensive nationwide figure of HCC in the Republic of Korea over the past 12 years. Second, data regarding second-line treatment modalities were incomplete. Therefore, we could not assess the beneficial role of sorafenib as a rescue therapy. Finally, since this study comprised HCC patients who were diagnosed from 2003 to 2014 in the Republic of Korea, the recent advances in the field of diagnosis and treatment for HCC cannot be reflected. For example, owing to the introduction of the Liver Imaging Reporting and Data System (LI-RADS), the more delicate differential diagnosis might be allowed [33,34] and not only newer interventional (e.g., radio-embolization) or radiotherapeutic (e.g., stereotactic body radiotherapy [35] and tomotherapy [36]) techniques but also second-line systemic chemotherapeutic agents (e.g., regorafenib and nivolumab) and other first-line agents (e.g., lenvatinib) become available nowadays. Therefore, such kinds of studies based upon the updated practice should be performed.

In conclusion, over the past 12 years, some changes in the baseline epidemiological characteristics of HCC were noted, and curative treatments became more widely available across early to advanced stages, generally improving overall prognosis. Despite sorafenib reimbursement, LRTs remain the mainstay of first-line treatment for BCLC stage C patients. Our analyses are a valuable reference for the establishment of priorities in HCC control, as well as the evaluation of the current situation in the Republic of Korea.

## Supporting information

S1 Table The summary of the S1 Fig. (DOCX)

S2 Table Predictors affecting the overall survival. (DOCX)

S1 Fig The detailed first-line treatment modalities according to BCLC stage in the E-Cohort (A) and L-Cohort (B). (TIF)

S2 Fig Kaplan-Meier analysis of overall survival according to treatment modalities (trans-hepatic arterial therapy vs. other LRTs) among BCLC stage B patients. (TIF)

S3 Fig Kaplan-Meier analysis of overall survival according to treatment modalities (sorafenib vs. LRTs) among BCLC stage C patients with Child-Pugh class A since 2008. (TIF)
