# Supplementary material for: Changes in real-life practice for hepatocellular carcinoma patients in the Republic of Korea over a 12-year period: A nationwide random sample study
Source: PLoS One. 2019 Oct 17;14(10):e0223678. doi: 10.1371/journal.pone.0223678 (PMC6797085; doi:10.1371/journal.pone.0223678)
Supplement: S1 Table — (DOCX) [file pone.0223678.s001.docx]

**S1 Table. The summary of the S1 Fig**

| **BCLC stages** | **Treatment modalities** | **E-Cohort** | **L-Cohort** |
| --- | --- | --- | --- |
| **0~A** | Curative treatment^a^ | 35.1% | 55.0% |
|  | Trans-hepatic arterial therapy^a^ | 53.1% | 39.1% |
|  | Systemic chemotherapy | 0.3% | 0.4% |
|  | External beam radiotherapy | 0.2% | 0.2% |
|  | Best supportive care^a^ | 3.0% | 4.6% |
|  | Unknown^a^ | 8.3% | 0.7% |
| **B** | Curative treatment^a^ | 11.4% | 23.2% |
|  | Trans-hepatic arterial therapy^a^ | 65.5% | 58.8% |
|  | Systemic chemotherapy^a^ | 0.8% | 2.0% |
|  | External beam radiotherapy | 0.2% | 0.8% |
|  | Best supportive care^a^ | 7.5% | 12.9% |
|  | Unknown^a^ | 14.6% | 2.3% |
| **C** | Curative treatment^a^ | 9.6% | 17.2% |
|  | Trans-hepatic arterial therapy^a^ | 48.3% | 41.6% |
|  | Systemic chemotherapy^a^ | 4.3% | 13.5% |
|  | External beam radiotherapy | 3.1% | 3.5% |
|  | Best supportive care^a^ | 13.1% | 21.1% |
|  | Unknown^a^ | 21.6% | 3.0% |
| **D** | Curative treatment | 10.1% | 12.9% |
|  | Trans-hepatic arterial therapy | 24.5% | 23.8% |
|  | Systemic chemotherapy^a^ | 1.3% | 3.6% |
|  | External beam radiotherapy | 0.8% | 1.7% |
|  | Best supportive care^a^ | 21.4% | 47.5% |
|  | Unknown^a^ | 41.9% | 10.5% |

**Abbreviations:** BCLC, Barcelona clinic liver cancer

^a^p<0.0
