# Supplementary material for: Changes in real-life practice for hepatocellular carcinoma patients in the Republic of Korea over a 12-year period: A nationwide random sample study
Source: PLoS One. 2019 Oct 17;14(10):e0223678. doi: 10.1371/journal.pone.0223678 (PMC6797085; doi:10.1371/journal.pone.0223678)
Supplement: S2 Table — (DOCX) [file pone.0223678.s002.docx]

**S2 Table. Predictors affecting the overall survival**

| **Variables** | **Univariate analysis** | **Multivariate analysis** | |
| --- | --- | --- | --- |
|  | **p-value** | **Adjusted HR** | **p-value** |
| **Age** | <0.001 | 1.0414 (1.012-1.017) | <0.001 |
| **Male gender** | <0.001 | 1.219 (1.152-1.289) | <0.001 |
| **Non-viral etiologies** | <0.001 | 0.991 (0.940-1.044) | 0.726 |
| **BCLC stages** |  |  |  |
| **0~A** | Reference | Reference | |
| **B** | <0.001 | 1.962 (1.827-2.106) | <0.001 |
| **C** | <0.001 | 3.314 (3.147-3.489) | <0.001 |
| **D** | <0.001 | 3.225 (2.957-3.217) | <0.001 |
| **Child-Pugh class B and C (vs. A)** | <0.001 | 2.129 (2.025-2.239) | <0.001 |
| **L-cohort (vs. E-cohort)** | <0.001 | 0.712 (0.679-0.748) | <0.001 |

**Abbreviations:** HR, hazard ratio; BCLC, Barcelona clinic liver cancer
