# Supplementary figures and images for: Changes in real-life practice for hepatocellular carcinoma patients in the Republic of Korea over a 12-year period: A nationwide random sample study
Source: PLoS One. 2019 Oct 17;14(10):e0223678. doi: 10.1371/journal.pone.0223678 (PMC6797085; doi:10.1371/journal.pone.0223678)

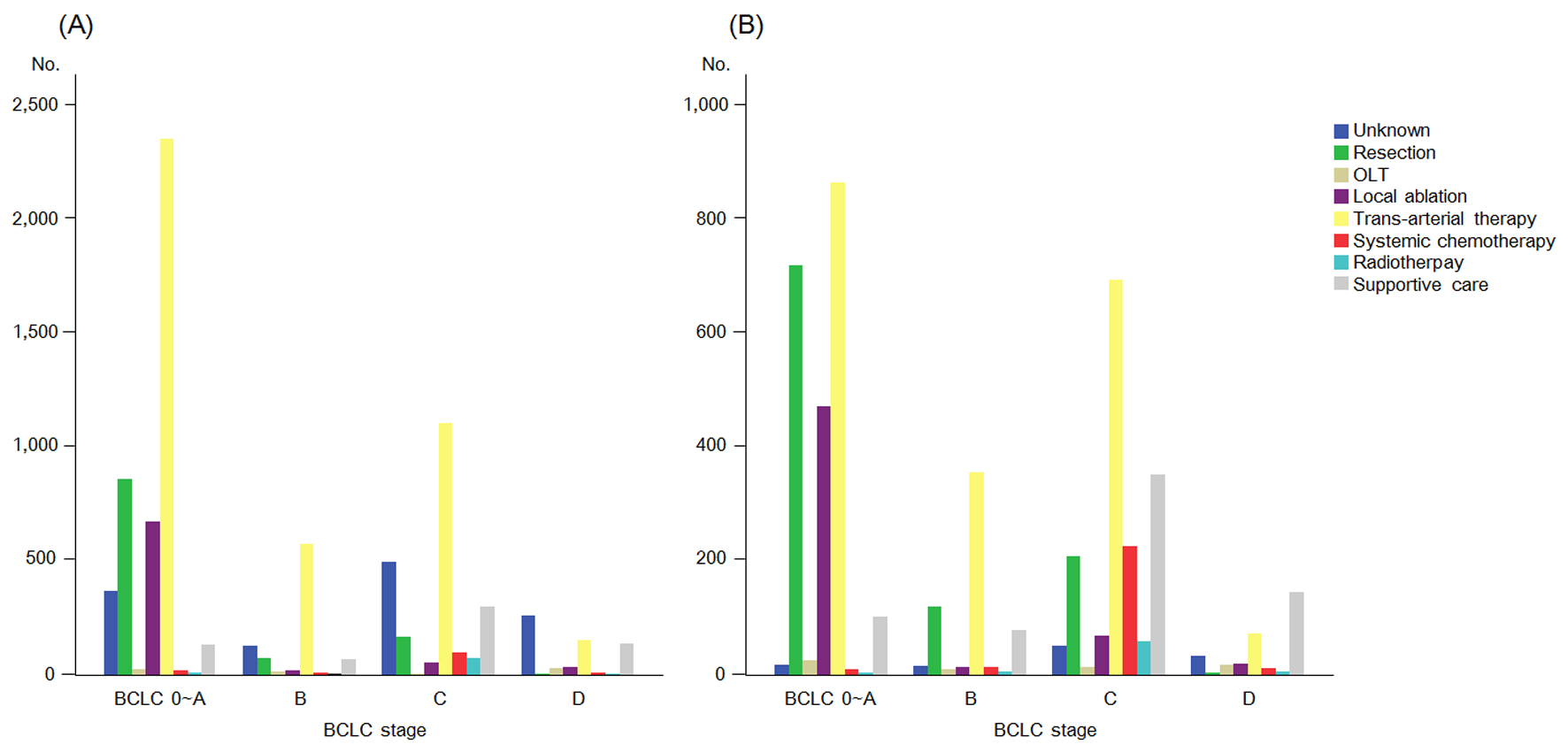

Supplement: S1 Fig — (TIF) [file pone.0223678.s003.tif]

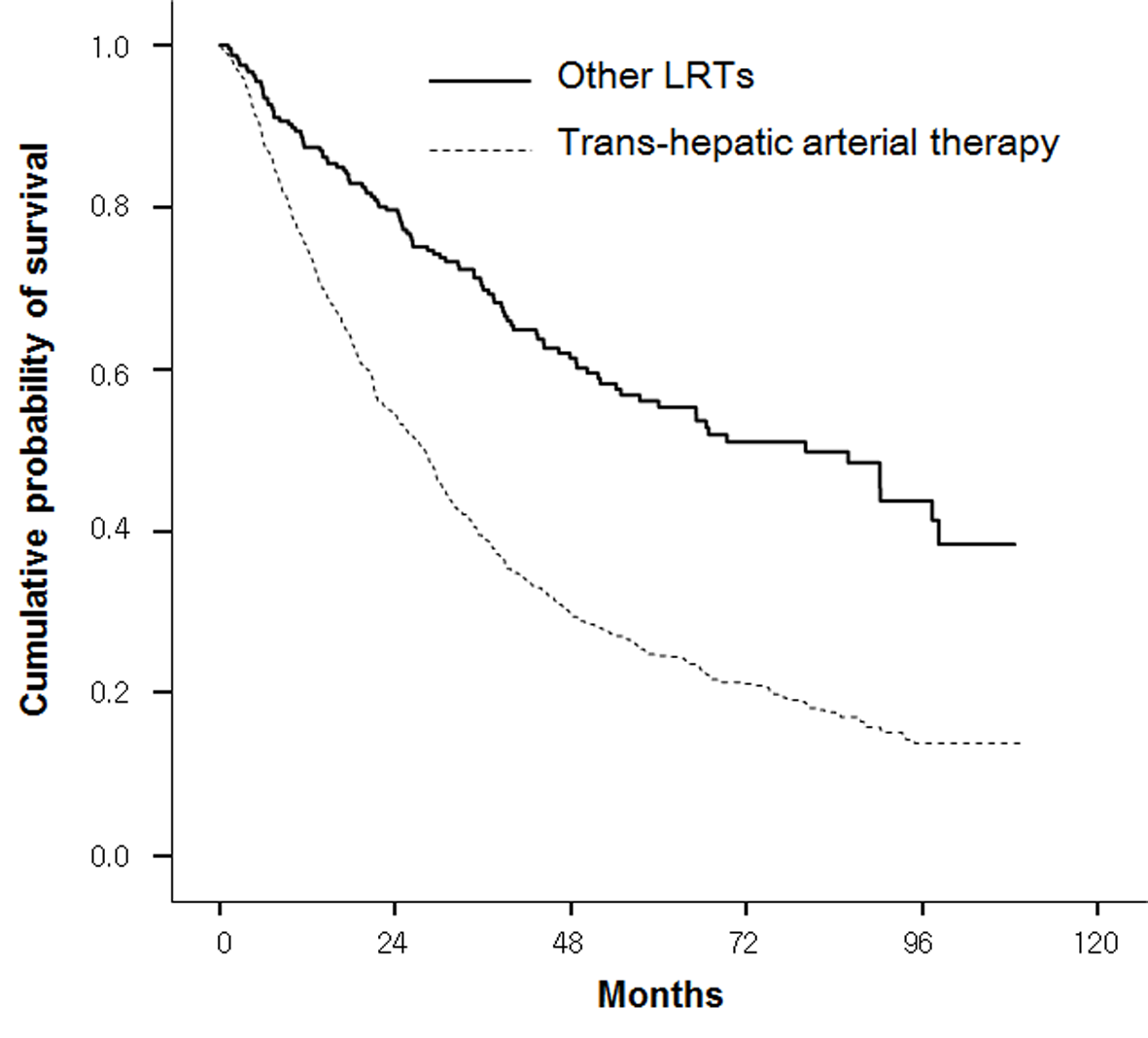

Supplement: S2 Fig — (TIF) [file pone.0223678.s004.tif]

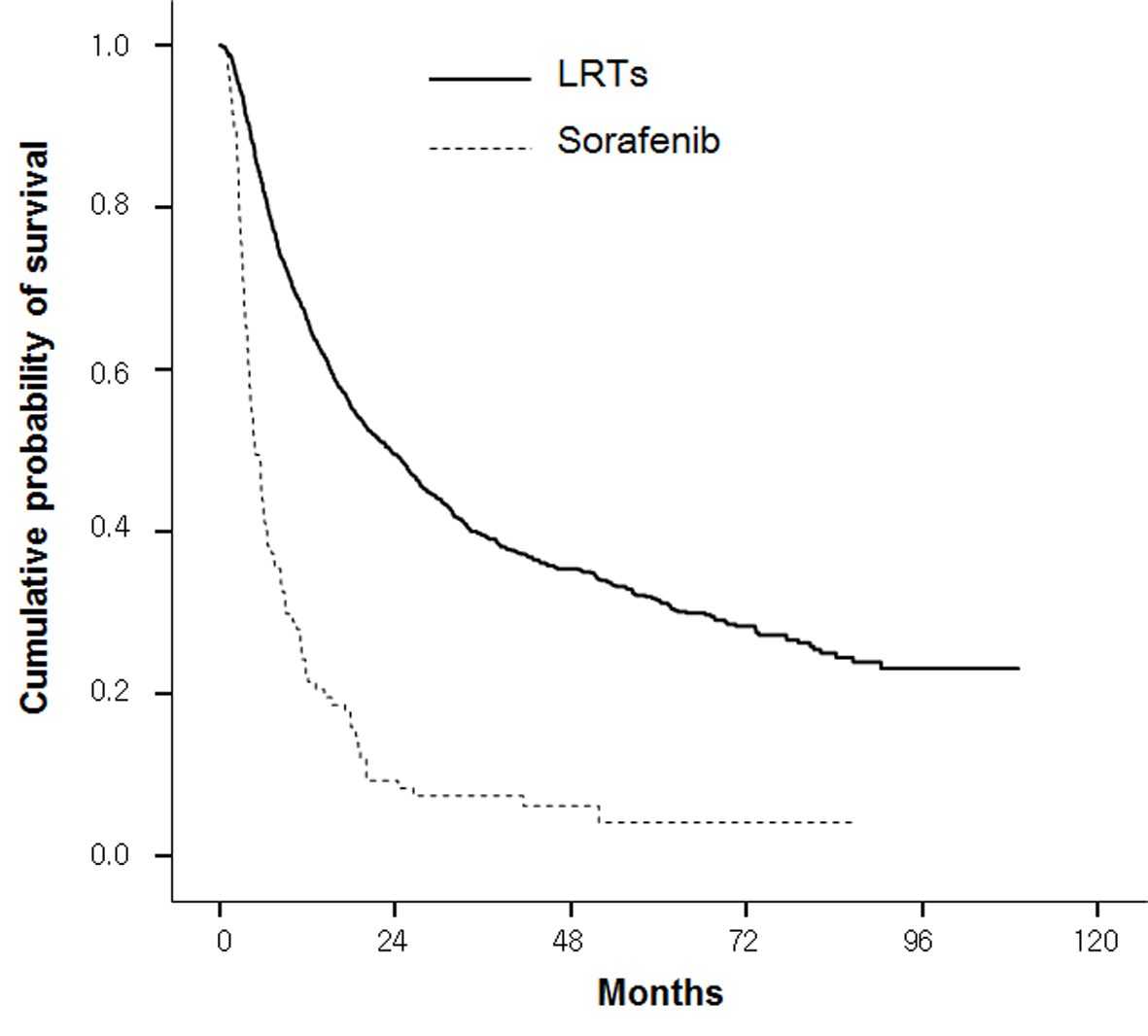

Supplement: S3 Fig — (TIF) [file pone.0223678.s005.tif]
